# Clozapine‐induced pulmonary embolism in a patient with no pre‐existing risk factors: A case report

**DOI:** 10.1002/ccr3.5497

**Published:** 2022-03-18

**Authors:** Jing Yi Kwan, Maha Noor

**Affiliations:** ^1^ Lynfield Mount Hospital Bradford District Care Trust Bradford UK

**Keywords:** antipsychotic, case report, clozapine, pulmonary embolism, schizophrenia, venous thromboembolism

## Abstract

We report the case of a 51‐year‐old Caucasian woman who developed a pulmonary embolism in the absence of any pre‐existing risk factors for VTE, 3 weeks following clozapine initiation for treatment resistant paranoid schizophrenia. She was initially misdiagnosed and treated for suspected COVID‐19 infection.

## Graphical abstract

This case highlights the need for a greater awareness amongst all clinicians regarding the association between antipsychotic therapy, particularly clozapine, and thrombotic events and the importance of regular VTE risk assessments in patients taking anti‐psychotic medication. Further research is warranted in order to specify the threshold level of risk at which pharmacological VTE prophylaxis should be commenced alongside anti‐psychotic therapy, even in low‐risk patients.

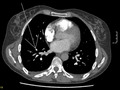

## 1 INTRODUCTION

Clozapine is a tricyclic dibenzodiazepine, second‐generation antipsychotic, typically reserved for people with treatment‐refractory schizophrenia, defined as two‐failed trials of other antipsychotics of adequate dose and duration.^1^ This is due to its association with a variety of adverse effects, including sedation, weight gain, diabetes mellitus, tachycardia, myocarditis, and seizures.^2^, ^3^ Due to the risk of neutropenia and agranulocytosis, blood test monitoring is also required.^2^, ^3^ Pulmonary embolism (PE) has been reported as a rare adverse effect of clozapine.^3^, ^4^

A PE occurs when a blood clot formed in the deep veins of the legs, (deep vein thrombosis, DVT) travels to the pulmonary vasculature via the venous system (venous thromboembolism), causing occlusion of blood flow and oxygenation in the lungs.^5^ The presence of one or more components of Virchow's triad—vein wall injury, hypercoagulability, and stasis of blood flow, increases the risk of developing venous thromboembolisms (VTEs).^6^ Established risk factors for VTE include advanced age, inherited coagulation abnormalities, acute medical illnesses, recent surgery, underlying malignancy, pregnancy, and estrogen containing medications.^6^

A growing number of studies have described an association between antipsychotic therapy, particularly clozapine,^7^, ^8^, ^9^ and an increased risk of VTEs.^10^, ^11^, ^12^, ^13^, ^14^, ^15^, ^16^ Several mechanisms have been proposed, such as venous stasis secondary to sedation and reduced mobility,^17^, ^18^ increased platelet adhesion and aggregation,^19^, ^20^ and elevated serum IgM antiphospholipid antibodies.^21^ Despite this, current UK guidelines do not routinely recommend pharmacological VTE prophylaxis alongside antipsychotic treatment.^22^

## 2 CASE HISTORY

A 51‐year‐old Caucasian female was admitted to an inpatient psychiatric unit presenting with new‐onset symptoms of auditory hallucinations accompanied with paranoid and hypochondriacal delusions. There were no negative symptoms including withdrawal, poverty of speech, or immobility. She had a past psychiatric history of depressive disorder, and a medication overdose attempt following separation with her partner five months earlier. She had no past medical history or family history of note and did not take any regular medications on admission. Her BMI was 22.7 kg/m^2^. She was a non‐smoker and social drinker, although had been abstinent from alcohol for the last few months. Her symptoms were associated with a major functional decline, having negatively affected her personal, occupational and psychosocial wellbeing. Consequently, a primary diagnosis of paranoid schizophrenia was established.

Commencement of olanzapine at 10 mg daily, titrated up to 20 mg daily, saw no resolution of symptoms over a four‐week period, while marked sedation occurred. Treatment was switched over to haloperidol, up titrated to 15 mg daily given for approximately 6 weeks. Significant extrapyramidal side effects ensued including akathisia and dystonia affecting the lower limbs, which were managed successfully with procyclidine. As her symptoms of auditory hallucinations and delusional ideation persisted despite receiving two antipsychotics at adequate doses and duration, a diagnosis of treatment‐resistant‐schizophrenia was made. Baseline investigations were obtained for clozapine initiation—a physical examination was unremarkable, serum analysis (Table 1) was within normal range aside from increments in cholesterol and triglycerides following antipsychotic therapy. An electrocardiogram (ECG) showed a normal sinus rhythm.

**TABLE 1:** Progression of blood results from pre‐clozapine routine bloods to hospital admission

	Pre‐clozapine initiation	Day 10 (monitoring)	Day 15 09:00 (monitoring)	Day 15 21:41	Day 18 Hospital Admission
Hemoglobin (115–160 g/L)	155	150	151	144	146
Platelet count (150–400 × 10^9^/L)	360	370	391	344	356
White cell count (4.00–11.00 × 10^9^/L)	6.85	7.10	10.84	9.94	10.90
Neutrophils (2.00–7.50 × 10^9^/L)	4.92	4.83	8.48	7.60	8.85
Lymphocyte (1.00–4.50 × 10^9^/L)	1.07	1.50	0.91	1.00	0.77
CRP (<10.0 mg/L)	<5.0	<5.0	23	81	197
Troponin (<37 ng/L)	2.6	2.8	<2.5	5	–
Coagulation Profile					
Prothrombin time (9–14 s)	–	–	–	–	11.8
INR (0.8–1.3)	–	–	–	–	1.0
APTT (23.5–37.5 s)	–	–	–	–	29.5
APTT ratio (0.8–1.0 s)	–	–	–	–	1.0
Fibrinogen derived level (1.5–5.9 g/L)	–	–	–	–	>5.9
D‐dimer (0–230 mg/L)	–	–	–	–	506
Urea and electrolytes					
Sodium (133–146 mM)	140	–	–	133	136
Potassium (3.5–5.3 mM)	4.5	–	–	4.1	4.3
Urea (2.5–7.8 mM)	5.6	–	–	5.1	5.1
Creatinine (45–84 µM)	67	–	–	69	65
eGFR (≥90)	80	–	–	88	>90
Liver function tests					
Bilirubin (2–21 µM)	10	–	–	–	–
ALT (iu/L <40)	13	–	–	–	–
Albumin (35–50 g/L)	40	–	–	–	–
Alkaline Phosphate (30–130 iu/L)	116	–	–	–	–
Thyroid function tests					
TSH (0.20–4.00 µ/L)	2.0	–	–	–	–
Free T4 (10.0–20.0 pM)	15.0	–	–	–	–
Prolactin (<600 µ/L)	215	–	–	–	–
HbA1c	33	–	–	–	–
Lipid Profile					
Cholesterol (<5 mM)	5.7	–	–	–	–
HDL Cholesterol (>1 mM)	1.3	–	–	–	–
Triglycerides (<2.3 mM)	2.6	–	–	–	–
LDL Cholesterol (<3 mM)	3.3	–	–	–	–
Cholesterol: HDL ratio (<4)	4.4	–	–	–	–

In accordance with the UK National Institute for Health and Care Excellence (NICE) guidelines for minimizing VTE risk in psychiatric inpatients,^23^ a VTE prophylaxis questionnaire was completed on admission and reviewed during clozapine therapy. The questionnaire, based on the UK Department of Health VTE risk assessment tool,^23^ included age > 60, dehydration, active cancer or undertaking cancer treatment, thrombophilia, obesity, significant medical comorbidities (heart disease, metabolic conditions, endocrine or respiratory pathologies, acute infection, inflammatory conditions), personal history or first degree relative with a history of VTE, use of hormonal replacement therapy (HRT) or estrogen containing contraceptive therapy, varicose veins with phlebitis, and pregnancy. It identified no risk factors for thromboembolic disease, thereby categorizing her as low risk.

Clozapine was initiated at 12.5 mg, doubled to 25 mg the following day, then up‐titrated with increments of 25 mg per day up to a dose of 200 mg a day, with the exception of maintenance at 100 mg for 3 days to monitor clinical status.

## 3 DIFFERENTIAL DIAGNOSIS, INVESTIGATIONS AND TREATMENT

**FIGURE 1:**
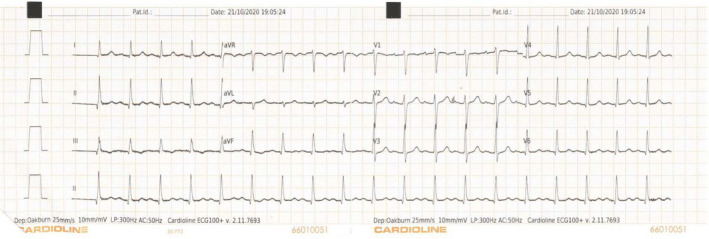
ECG demonstrating sinus tachycardia with no other abnormalities

Within the first 14 days, the patient developed several known adverse effects of clozapine including hypersalivation, sedation, constipation, lethargy, and tachycardia (100–120 beats per minute).^2^, ^3^ She was investigated for clozapine‐induced‐myocarditis, though successive ECGs exhibited sinus tachycardia only (Figure 1), while troponin and C‐reactive protein (CRP) levels remained normal (Table 1). The patient also exhibited symptoms of a persistent dry cough. Due to the current pandemic, COVID‐19 infection was considered, however, three consecutive COVID swabs returned as negative. From a psychiatric point of view, some resolution of auditory hallucinations was reported by the patient, along with intermittent insight toward her delusional ideation.

On Day 15, monitoring bloods revealed a rise in neutrophils (8.48 × 10^9^/L), monocytes (0.88 × 10^9^/L), and CRP (23 mg/L), without an obvious source of infection. Later that night, she developed symptoms of coryza, fever, and left‐sided pleuritic chest pain. Vital parameters demonstrated a heart rate (HR) of 136, respiratory rate (RR) of 18, oxygen saturation at room air of (SpO_2_) 95%, blood pressure (BP) 132/78, and temperature of 37.8°C. Clinical examination revealed normal chest sounds with no evidence of wheeze or crepitations, alongside normal heart sounds with no added murmurs. There was no peripheral edema, and both calves were soft and non‐tender. An abdominal examination was unremarkable. Sequential electrocardiograms (ECG) displayed sinus tachycardia with no other changes, while repeat blood analysis revealed a CRP rise to 81 mg/L and normal troponin of 5. Urinalysis was unremarkable. Paramedical team assessed her for transfer to the medical hospital; however, her vital signs returned to normal, and symptoms of chest pain resolved. Owing to her presentation of marginal pyrexia, dry cough, and the disease prevalence, a second COVID‐19 swab was obtained, which also returned negative. Subsequently, an oral course of amoxicillin 500 mg four times a day for 7 days was initiated for a suspected lower respiratory tract infection (LRTI).

**FIGURE 2:**
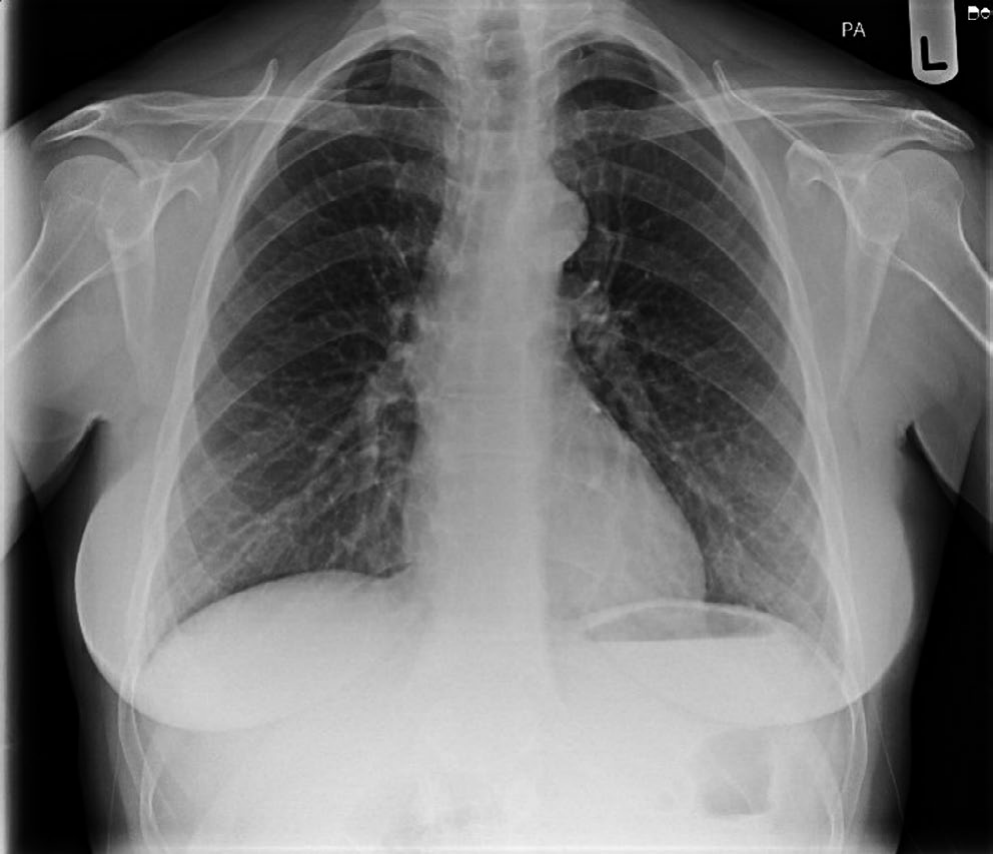
Chest X‐ray displaying no significant airspace pathology

**FIGURE 3:**
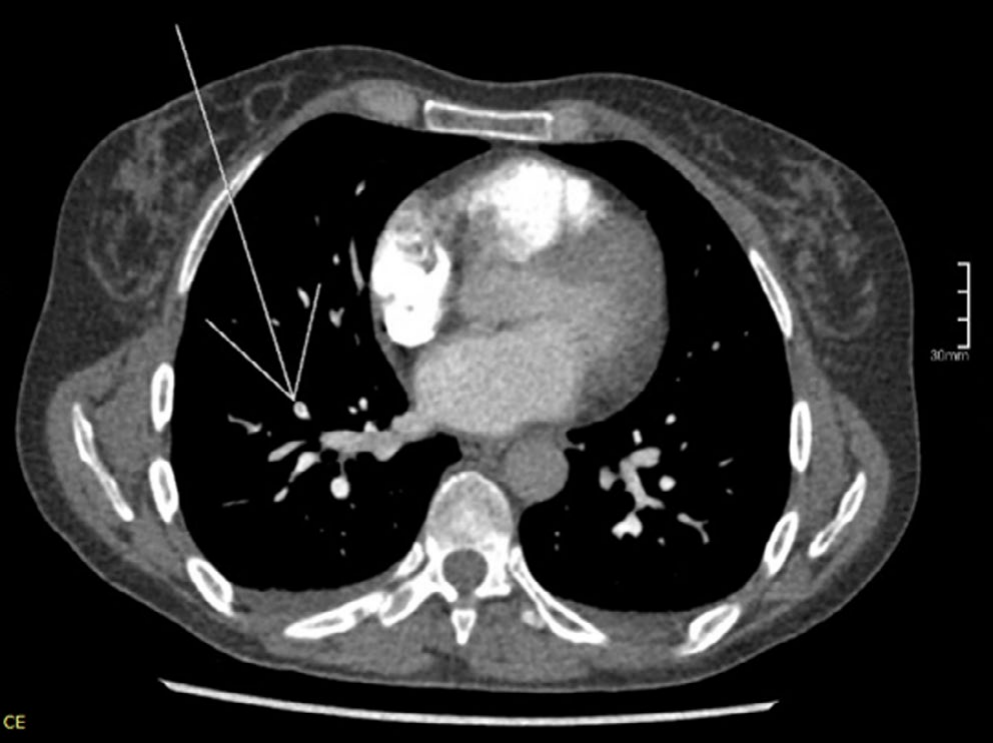
CTPA showing a subsegmental pulmonary embolism in the lateral branch of the right lower lobe

Following a brief period of recovery, on Day 18 she deteriorated again, with HR of 150, RR 24, SpO_2_ 97%, BP 92/46, and temperature of 38.7°C. She was admitted to the medical hospital for suspected respiratory sepsis ±COVID infection. An arterial blood gas displayed pH 7.43 (7.35–7.45), pO2 6.0 kpa (11.0–13.0), pCO2 4.2 kPa (4.7–6.0), and bicarbonate 20.5 (22.0–26.0). Serum analysis exhibited lymphopenia, (0.77 × 10^9^/L) along with raised neutrophils (8.85 × 10^9^/L), CRP (197 mg/L), and D‐dimer (506 mg/L), with an otherwise normal coagulation profile (Table 1). A Chest X‐ray exhibited no airspace pathology (Figure 2). As lymphopenia is associated with COVID‐19,^24^ a third COVID swab was obtained which also returned negative. An echocardiogram showed normal ventricular size and function bilaterally with no abnormalities, thus ruling out myocarditis. Consequently, a computerized tomography pulmonary angiogram (CTPA) was arranged, which demonstrated a subsegmental pulmonary embolism in the lateral branch of the right lower lobe with no evidence of right heart strain (Figure 3).

The patient was admitted to the medical unit for a total of 6 days. Initial management consisted of oxygen therapy and intravenous cefotaxime and clarithromycin for LRTI. The diagnosis of pulmonary embolism led to the initiation of rivaroxaban at a dose of 15 mg twice daily for 21 days, followed by 20 mg once a day for the remainder of a 6‐month course. The patient had no history of unintentional weight loss, night sweats, bruising or infections, nor any hematological evidence of malignancy, and a breast examination was normal. Due to the absence of any relevant clinical signs or symptoms, further imaging for an underlying cancer was not arranged as per recommendation of the NICE guidelines on the management of venous thromboembolic disease.^25^

## 4 OUTCOME AND FOLLOW‐UP

Clozapine was discontinued immediately at the psychiatrist's discretion and patient's request, and aripiprazole was initiated due to its lower metabolic adverse effect profile.^26^ Despite titration to 25 mg once daily given for a seven‐week period, no improvement in psychotic symptoms occurred. Amisulpride had a similar outcome. Successful resolution of both auditory hallucinations and delusional ideation has been noted since the commencement of flupentixol 3 mg daily. No further physical health concerns were expressed by the patient.

## 5 DISCUSSION

In recent years, increasing evidence has emerged regarding an association between antipsychotic therapy and venous thromboembolisms.^6^, ^9^, ^10^, ^11^, ^12^, ^13^ A study of the World Health Organization (WHO) database for adverse drug reactions revealed a strong association between venous thromboembolisms and second‐generation antipsychotics including clozapine, olanzapine, sertindole, and zuclopenthixol.^10^ A large US hospital database of 28,723,711 adults in 2006 demonstrated that the risk of PE was greater in antipsychotic users than in the total population (*p* < 0.001), and clozapine in higher doses was associated with the greatest risk amongst all antipsychotics.^11^ Meta‐analyses of observational studies have also reported significantly increased risks of PE in antipsychotic users, particularly in previously antipsychotic‐naive patients.^14^, ^15^, ^16^ Therefore, it is important to be aware of this rare but significant adverse effect.

Clozapine‐induced pulmonary embolisms are associated with a high mortality rate. In 1997, Walker et al illustrated that current clozapine exposure related to a five‐fold increase in risk of fatal PE compared with past exposure to clozapine.^27^ A more recent systematic review of the literature demonstrated a mortality rate of 36.36%^7^ whilst a case series by Sarvaiya et al showed a mortality rate of 26.1% with 6 patients out of 23 cases dying on presentation.^28^ This demonstrates that although PE is a rare adverse effect, it is a highly lethal one.

The mechanisms by which antipsychotics including clozapine‐induced venous thromboembolisms are multifactorial. Significant sedation and an average weight gain of 7–11 kg are affiliated with clozapine therapy.^17^ This contributes to reduced mobility and venous stasis,^17^, ^18^ thereby increasing the risk of developing VTEs. Our patient did not have any objective evidence of weight gain throughout her admission. She remained freely mobile, engaging in twice weekly walks of the hospital grounds with the occupational therapy team. Clozapine has also been found to increase platelet adhesion and aggregation and reduce activated partial thromboplastin time (aPTT),^19^ which can lead to the development of VTE. A recent in vitro study revealed that in a dose‐dependent manner, clozapine affects fibrin formation by reducing coagulation speed and fibrin fiber thickness, leading to fibrinogen acquiring increased thrombogenic properties.^20^ Treatment with clozapine for a duration longer than 2½ years can lead to elevated serum concentrations of IgM antiphospholipid antibodies, which are, although rarely, associated with arterial and venous thrombosis.^21^ Our patient had commenced clozapine only 14 days prior, and while anti‐phospholipid antibody levels were not evaluated in our patient, serum analysis showed a normal APTT (Table 1).

A pulmonary embolism occurred in our patient at a clozapine dose of only 200 mg a day. This is considerably lower than the standard maintenance dose of 300–600 mg typically established in treatment of schizophrenia.^28^ In a systematic review of 23 case reports, it was found that clozapine associated pulmonary embolisms can be early onset and dose independent—therefore, pulmonary embolisms can occur even at lower than usual doses of clozapine.^29^ These findings should prompt regular VTE risk monitoring and consideration of prophylactic treatment for VTE in patients on clozapine therapy. While the UK NICE guidelines acknowledge an increased risk of VTE with antipsychotic therapy, the difference in risk between individual antipsychotics remains indeterminate.^22^ Therefore, prophylaxis is not routinely recommended. In psychiatric inpatients, the assessment of VTE risk on admission and following any change in clinical change has been recommended.^23^ Although “consideration” of pharmacological VTE prophylaxis is advised in patients for whom VTE risk outweighs bleeding risk,^23^ the threshold for this consideration is not specified, particularly for low patients newly commenced on antipsychotic therapy.

There is a paucity of published evidence investigating the potential benefits and risks of pharmacological VTE prophylaxis in patients on antipsychotic therapy. One recent study investigating the risk of VTE recurrence in patients newly commenced on antipsychotics showed that the use of antipsychotic agents among patients with a first episode of VTE and full‐dose anticoagulation was not associated with an increased risk of recurrent thromboembolic events.^30^ This might suggest some benefit of pharmacological prophylaxis in the setting of antipsychotic therapy use, provided there is no increased risk of bleeding.

One clinical challenge encountered in this case relates to the variation in clinical presentation of sub‐segmental pulmonary embolisms (SSPE) as compared with central pulmonary embolisms. While central PEs most commonly present with dyspnea, patients with SSPEs may experience chest pain or be completely asymptomatic.^31^ The reduced embolic burden in SSPEs results in less hypoxia, less hemodynamic instability, lower plasma D‐dimer and troponin, and less concurrent proximal DVT.^31^ Consequently, pre‐test clinical prediction scores for pulmonary embolism (e.g., the Wells score) are less sensitive in SSPE.^31^ Our patient experienced symptoms of dry cough, fever, lethargy, and chest pain which resolved shortly afterward, in the absence of clinical signs and symptoms of DVT, ECG changes, or any established risk factors for PE. At initial presentation, she had a Wells score of 1.5 for HR >100, which is classified as low risk for PE. The delay in diagnosis of PE was partially because she was repeatedly screened for myocarditis—a more recognized side effect of clozapine, and COVID‐19 prevalence—which has a similar respiratory presentation to PE. Therefore, a greater awareness of VTE potential even in low‐risk patients can prevent delays in diagnosis and treatment, improving morbidity, and mortality in patients taking clozapine.

It is important to highlight that our patient would have been classified as low risk by any VTE risk stratification system available, which signifies the clinical challenge posed by such cases. Although review of guidelines and literature regarding antithrombotic prophylaxis alongside clozapine is important, more crucial is the recognition that clozapine‐induced VTEs are dose independent and can occur early on during treatment. They can occur in patients with minimal/no risk factors and can be highly lethal. Therefore, clinicians need to have an enhanced awareness of the possibility of thrombotic events in patients on antipsychotics including clozapine. This is especially true in the current era of COVID.

To conclude, we recommend that when prescribing clozapine, clinicians should undertake regular reviews of VTE risk, and encourage patients to ambulate and maintain a healthy weight. The threshold for consideration of prophylactic antithrombotic treatment especially where additional risk factors are present should be low.

## CONFLICT OF INTERESTS

The authors declare that they have no competing interests.

## AUTHOR CONTRIBUTION

All authors (JK and MH) contributed equally to this work and share first authorship.

## CONSENT

Written informed consent was obtained from the patient to publish this report in accordance with the journal's patient consent policy.

## Data Availability

The data that support the findings of this study are available from the corresponding author upon reasonable request.
